# Hypermethylation and loss of retinoic acid receptor responder 1 expression in human choriocarcinoma

**DOI:** 10.1186/s13046-017-0634-x

**Published:** 2017-11-23

**Authors:** H. Huebner, R. Strick, D. L. Wachter, S. Kehl, P. L. Strissel, R. Schneider-Stock, A. Hartner, W. Rascher, L. C. Horn, M. W. Beckmann, M. Ruebner, F. B. Fahlbusch

**Affiliations:** 1 Department of Gynaecology and Obstetrics, University Hospital Erlangen, Friedrich-Alexander University Erlangen-Nuremberg, Comprehensive Cancer Center Erlangen-EMN, Erlangen, Germany; 2 Institute of Pathology, University Hospital Erlangen, Friedrich-Alexander University Erlangen-Nuremberg, Comprehensive Cancer Center Erlangen-EMN, Erlangen, Germany; 3 Department of Pediatrics and Adolescent Medicine, University Hospital Erlangen, Friedrich-Alexander University Erlangen-Nuremberg, Loschgestraße 15, 91054 Erlangen, Erlangen, Germany; 4 Division Molecular Pathology, Institute of Pathology, University of Leipzig, Leipzig, Germany

**Keywords:** Placenta, RARRES1, Retinoic acid, Choriocarcinoma, DNA methylation, TIG1, Epigenetic

## Abstract

**Background:**

Human placental development resembles tumorigenesis, due to the invasive and fusogenic potential of trophoblasts. However, these features are tightly controlled in trophoblasts. Disturbance of this spatial and temporal regulation is thought to contribute to the rare formation of choriocarcinomas. Promoter hypermethylation and loss of the tumor suppressor *Retinoic acid receptor responder 1* (*RARRES1*) were shown to contribute to cancer progression. Our study investigated the epigenetic and transcriptional regulation of *RARRES1* in healthy human placenta in comparison to choriocarcinoma cell lines and cases.

**Methods:**

Three choriocarcinoma cell lines (Jeg-3, JAR and BeWo) were treated with three different retinoic acid derivates (Am580, Tazarotene and all-trans retinoic acid) and 5-aza-2′-deoxycytidine. We analyzed *RARRES1* promoter methylation by pyrosequencing and performed realtime-PCR quantification to determine *RARRES1* expression in placental tissue and trophoblastic cell lines. Additionally, RARRES1 was stained in healthy placentas and in biopsies of choriocarcinoma cases (*n* = 10) as well as the first trimester trophoblast cell line Swan71 by immunofluorescence and immunohistochemistry.

**Results:**

In the choriocarcinoma cell lines, *RARRES1* expression could not be induced by sole retinoic acid treatment. Stimulation with 5-aza-2′-deoxycytidine significantly induced *RARRES1* expression, which then could be further increased with Am580, Tazarotene and all-trans retinoic acid. In comparison to healthy placenta, choriocarcinoma cell lines showed a hypermethylation of the *RARRES1* promoter, which correlated with a reduced *RARRES1* expression. In concordance, RARRES1 protein expression was lost in choriocarcinoma tissue. Additionally, in the trophoblastic cell line Swan71, we found a significant induction of *RARRES1* expression with increased cell density, during mitosis and in syncytial knots.

**Conclusions:**

Our findings showed that *RARRES1* expression is absent in choriocarcinoma due to promoter methylation. Based on our analysis, we hypothesize that RARRES1 might exert tumor suppressive functions in multiple cellular processes (e.g. cell cycle regulation, adhesion, invasion and apoptosis).

**Electronic supplementary material:**

The online version of this article (10.1186/s13046-017-0634-x) contains supplementary material, which is available to authorized users.

## Background

Human hemochorial placentation closely resembles processes otherwise seen in cancer. The capacity of trophoblasts to migrate and invade the maternal decidua, as well as their ability to form the syncytiotrophoblast (SCT) via cell-fusion, shares high similarities with tumor cells [1]. As a consequence, trophoblasts are often characterized to be of pseudo-malignant nature. Not surprisingly, expression patterns of tumor suppressor genes in cancer are similar to those found in placenta [2, 3]. Recently, retinoic acid receptor responder 1 (*RARRES1*), also known as Tazarotene-induced gene 1 (*TIG1*), was identified as an important tumor suppressor gene [4, 5]. It was initially described as a novel retinoic acid (RA) receptor (*RARβ* and *γ*) regulated gene in skin graft cultures [6]. *RARRES1* is located on chromosome 3q25 and was reported to be one of the most commonly methylated loci in multiple cancers [7–9]. Epigenetic silencing of *RARRES1* expression and its effect on tumor cell invasion, proliferation and survival further underscored its tumor suppressive properties [10]. In prostate cancer an association between *RARRES1* hypermethylation and worse clinical outcome was reported [8].

Recently, we identified RARRES1 in the human placenta and determined its regulation by RA derivates. In the placenta, uncontrolled trophoblast growth result in malignant transformation as observed in choriocarcinomas [11].

Thus, in the light of previous reports regarding the role of epigenetic regulation of tumor suppressor genes in the human placenta [12, 13], we investigated the epigenetic and transcriptional regulation of the tumor suppressor *RARRES1* in the human placenta, choriocarcinoma cell lines and biopsies, as well as its potential functional properties.

## Methods

### Patient and tissue collection

Human term placentas (third trimester) were obtained from healthy patients (*n* = 74) after elective Caesarean section and processed within 1 h. The clinical data were summarized in Additional file 1: Table S1. After removal of the basal plate and chorionic membrane, a biopsy was obtained near the cord from every placenta. Placental tissues were snap frozen in liquid nitrogen and stored at −80 °C until further use. Formalin-fixed and paraffin-embedded (FFPE) sections were supplied by the Institute of Pathology, University Hospital Erlangen. Handling of patients and tissues was approved by the Ethics Committee at the University Erlangen-Nuremberg (No. 2180). A written informed consent was obtained from all participants. FFPE sections of 10 choriocarcinoma cases were provided by the Institute of Pathology of the University Hospital Leipzig. Five cases were intramolar and five pure choriocarcinomas.

### Immunohistochemical (IHC) staining

Human FFPE placental sections were deparaffinized and rinsed in a series of descending ethanol concentrations. IHC stains were performed on tissue sections using the LSAB + HRP kit (Agilent, Hamburg, Germany) according to the manufacturer’s instructions. Anti-human RARRES1 (ab92884, Abcam, Cambridge, United Kingdom, 1:6000) antibody was used. Nuclei were counter-stained by hematoxylin.

### Immunofluorescence (IF) staining

Human FFPE placental sections were deparaffinized and rehydrated as stated above. Slides were blocked using 1% bovine serum albumin in Tris-buffered saline (BSA/TBS) for 30 min at room temperature. Anti-human RARRES1 antibody was diluted in 1% BSA/TBS (1:400) and slides were incubated at 4 °C over-night. As secondary antibody Alexa Fluor 488 Donkey Anti-Mouse IgG (H + L) (Molecular Probes, Eugene, USA) was used at a dilution of 1:400 in 1% BSA/TBS and slides were incubated for 2 h at room temperature. Staining of the nuclei was performed using DAPI (Thermo Fischer, Darmstadt, Germany).

### Villous cytotrophoblast isolation

Villous cytotrophoblasts (VT) from healthy placentas of 15 different individuals were isolated using the Trypsin-DNase-Dispase/Percoll method, as previously described [14]. Before DNA and RNA extraction the fractionated trophoblasts were cryopreserved in liquid nitrogen. By fluorescence activated cell sorting (FACSCalibur, BD Biosciences) we could determine a percentage of 86.6–90% of the fractionated cells being trophoblastic [14].

### Cell culture

The choriocarcinoma cell lines BeWo, Jeg-3 and JAR were cultured under the following conditions: BeWo was grown in DMEM:F12 phenol-red free (high Glucose; Thermo Fischer, Darmstadt, Germany) supplemented with 10% fetal calf serum (FCS, Thermo Fischer, Darmstadt, Germany), 100 U/ml penicillin and 100 μg/ml streptomycin (1% P/S, Sigma-Aldrich, Taufkirchen, Germany). Jeg-3 cells were cultured in phenol-red free DMEM:F12 (Thermo Fischer, Darmstadt, Germany) supplemented with 10% FCS, 1% P/S and JAR cells in RPMI 1640 media (Thermo Fischer, Darmstadt, Germany) with 10% FCS, 10 mM Hepes (Sigma-Aldrich, Taufkirchen, Germany), 2 mM L-Glutamin (Sigma-Aldrich, Taufkirchen, Germany) and 0.1 mM non-essential amino acids (NEAA, Sigma Aldrich, Taufkirchen, Germany). The first trimester cell line Swan71 was a kind gift of Prof. G. Mor, Department of Obstetrics, Gynaecology and Reproductive Sciences, Reproductive Immunology Unit, Yale University School of Medicine, USA [15]. The cell line was grown in phenol red free DMEM:F12 with 10% FCS, 1%P/S and 0.1 mM NEAA.

For stimulation experiments cells were seeded at a density of 3x10^4^/ml (Swan71, Jeg-3 and JAR) or 4x10^4^/ml (BeWo) in a 12-well cell culture plate. Cell lines were cultivated in medium supplemented with 2.5% (Swan71) or 5% (Jeg-3, JAR, BeWo) charcoal treated fetal bovine serum (CTS, Thermo Fisher, Darmstadt, Germany) 24 h prior to and during stimulation. Cells were treated with 1.0 μM all-trans-retinoic acid (ATRA, Biomol, Hamburg, Germany), Tazarotene (TAZA, Sigma-Aldrich, Taufkirchen, Germany) or 200 nM Am580 (Tocris, Lille Cedex, France) for 24 to 72 h. In order to induce global DNA demethylation, the cell lines were additionally treated with 0.5 or 1.0 μM 2′-deoxy-5-azacytidine (AZA, Sigma-Aldrich, Taufkirchen, Germany) for 72 h.

For the analysis of RARRES1 expression in dependence of cell density, Swan71 and Jeg-3 cells were seeded at 0.75, 1.50, 3.0, 6.0, 12.0 and 18.0x10^4^ cells/ml in 12-well dishes. After 48 h cells were harvested for RNA isolation (Additional file 2: Figure S1). Jeg-3 cells were additionally treated with AZA for 72 h before harvesting.

### Genomic DNA extraction of tissues and cultivated cell lines

Genomic DNA was isolated from cultivated cell lines or 50–100 mg placental tissues as previously described in detail [16]. DNA was dissolved in 0.01% DEPC water.

### Bisulfite treatment and PCR amplification

Bisulfite treatment of 0.5–2.0 μg genomic DNA of placental tissues and trophoblast-like cell lines was performed using the EpiTect Bisulfite Kit (Qiagen, Hilden, Germany) according to the manufacturer’s instructions. Converted DNA (100 ng) was amplified using a thermal cycler (Thermo Fischer, Darmstadt, Germany) and the HRM-Mastermix (Qiagen, Hilden, Germany). Bisulfite treated DNA was used in a 25 μl reaction volume with 10 μM of both the forward and the reverse primer (Additional file 3: Table S2). PCR conditions were 95 °C for 15 min, 50 cycles of denaturation (30s at 95 °C), annealing (30s at 50 °C for the CpG region 1 and 55 °C for region 2) and extension (30s at 72 °C), followed by a final extension period of 7 min at 72 °C. Successful conversion and amplification were controlled using capillary electrophoresis (QIAxcel, Qiagen, Hilden, Germany).

### Pyrosequencing

Pyrosequencing (PyroMark Q24, Qiagen, Hilden, Germany) was performed according to the manufacturer’s instructions using PyroMark Gold Q24-reagents (Qiagen, Hilden, Germany). PCR and sequencing primer were designed using the PSW Assay design software 1.0.6 (Biotage, Sweden) as well as the online program MethPrimer [17]. 75 pmol of the respective CpG region specific sequencing primer was used (Additional file 3: Table S2). Each CpG region of interest was split into two sections and analyzed separately to obtain reproducible results. The second section was sequenced using serial pyrosequencing according to Tost et al. (2006) [18]. The methylation pattern was quantified by the PyroMark Q24-CpQ-Assay (Qiagen, Hilden, Germany). Correct sequencing was monitored using the EpiTect-PCR-Control-DNA set including methylated, unmethylated and unconverted control DNA. Data were analyzed by the PyroMark Q24 software.

### RNA extraction and cDNA synthesis

RNA was isolated from third trimester placental tissues as well as cultivated cell lines using peqGOLD TriFast (PEQLAB, Erlangen, Germany), as previously described [16]. Isolated RNA was treated with DNase I (Roche, Mannheim, Germany) to avoid DNA contamination during expression analysis. The High-Capacity-cDNA-Reverse-Transcription kit (Thermo Fischer, Darmstadt, Germany) was used to generate cDNA in a thermal cycler (ABI2720) for 2 h at 37 °C.

### Quantitative Realtime PCR (qRT-PCR)

Quantification of *RARRES1* mRNA expression in placental tissues and cell lines was achieved by qRT-PCR analysis as previously described [14]. In short, 10 nM forward and reverse primers and 40 ng cDNA were used to analyze the amplification of *RARRES1* via SYBR-green based technology (SYBR Select Master Mix, Thermo Fischer, Darmstadt, Germany). The expression was normalized using 18SrRNA and GAPDH as reference genes. Due to lack of differences, 18SrRNA data are presented only. Primer sequences are listed in Additional file 3: Table S2.

### Statistical analysis

All data are presented as mean ± standard error of the mean (SEM), which was calculated using Microsoft Excel 2010 (Microsoft Corporation, Redmond, Washington, USA). Differences between the subgroups were analyzed using the Mann-Whitney U-test (SPSS, IBM Inc., Nuremberg, Germany). Significances were adjusted using post hoc Bonferroni testing. *P*-values of less than 0.05 were considered as statistically significant.

## Results

### *RARRES1* promoter hypermethylation and reduced gene expression in choriocarcinoma cell lines

**Fig. 1:**
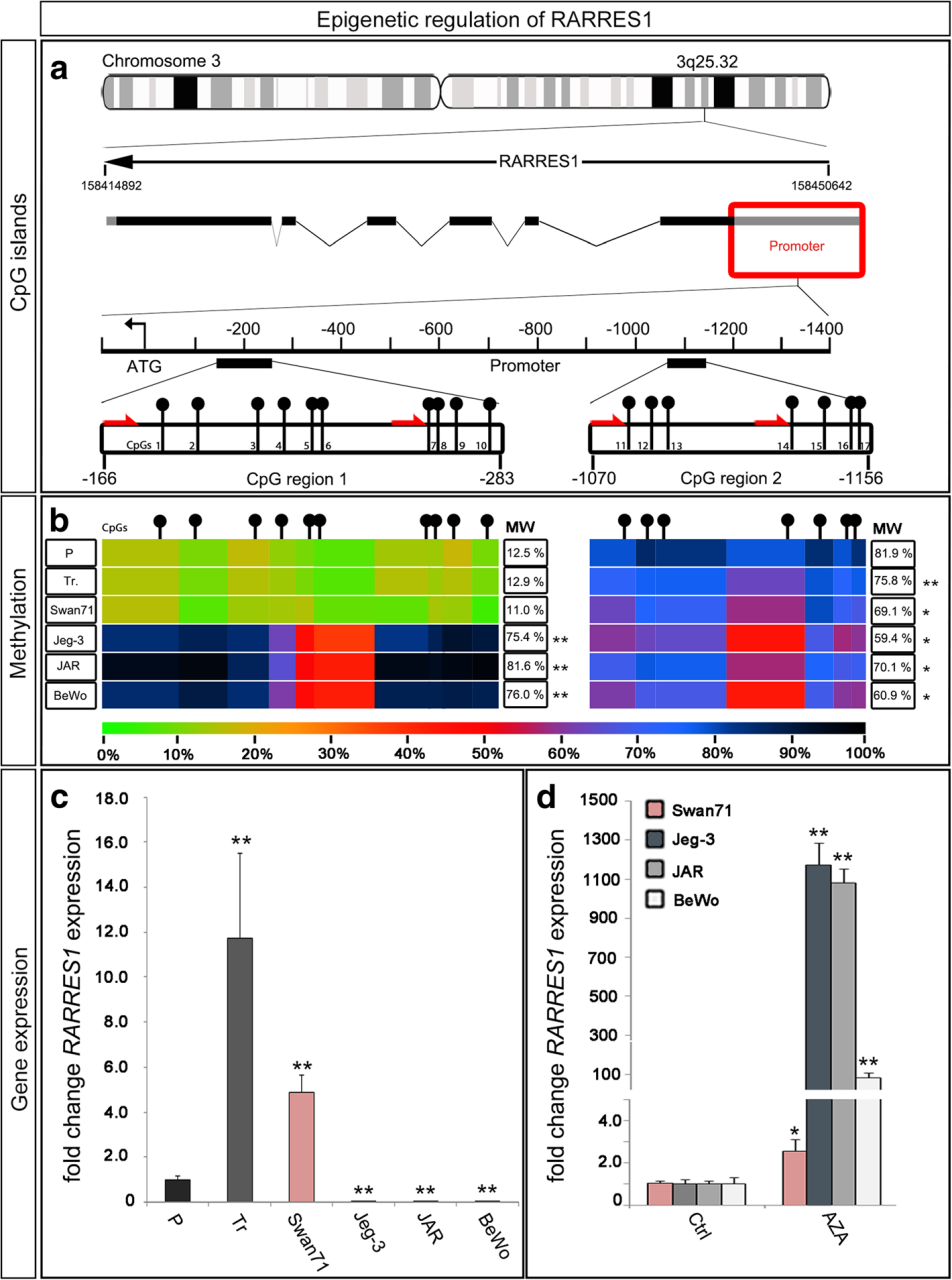
*RARRES1* methylation and expression pattern. **a** Gene locus of *RARRES1* on chromosome 3. The ideogram shows the *RARRES1* locus on 3q25.32. The genetic region is drawn schematically with the coding regions as black squares. Two CpG regions within the promoter were selected for further analysis. CpGs are marked with *black pins*. *Red arrows* symbolize the sequencing primers for the pyrosequencing reaction. **b** Heat-map for the promoter methylation pattern. DNA of 3rd trimester placentas (P; from *n* = 74 individuals), 3rd trimester trophoblasts (Tr; from *n* = 15 individuals), the trophoblast-like cell line Swan71 (n = 1) and the choriocarcinoma cell lines Jeg-3, JAR and BeWo (n = 1 each) were analyzed using pyrosequencing. The methylation intensity was illustrated by heat map coloring as indicated by the scale below, where green represents the unmethylated state (0%) and *black* the completely methylated state (100%). **c** Gene-expression analysis of RARRES1 in placental tissues (from *n* = 10 individuals), primary trophoblasts (from *n* = 5 individuals) as well as Swan71 cells and the choriocarcinoma cell lines (*n* = 9). Gene expression is indicated as relative to *RARRES1* expression of 3rd trimester placentas (P). **d** QRT-PCR analysis of AZA-treated cell lines relative to control (*n* = 9). Significance is marked by asterisks (* *p* < 0.05, ** *p* < 0.005). Data represent mean ± SEM

The *RARRES1* gene is localized on Chromosome 3q25.32 (Fig. 1a). To investigate the methylation pattern of the *RARRES1* promoter in placental tissues and cell lines, we performed pyrosequencing of two different CpG regions within the promoter. We chose an ATG-proximate (CpG region 1) and distal region (CpG region 2) of the size of about 100 bp each. These selected CpG regions are part of a CpG island, which was reported to be hypermethylated in prostate cancer and to contain several putative retinoic response elements [19]. CpG region 1 is 115 bp long and starts 166 bp upstream of the ATG codon. This region includes 10 CpG positions. The CpG region 2 lies about 900 bp further upstream, containing seven CpGs (Fig. 1a, black pins). Pyrosequencing of the *RARRES1* promoter revealed a hypomethylation of CpG region 1 (0–20% methylation) compared to region 2 (60–90% methylation) for placental tissues, isolated trophoblasts and the Swan71 cells (Fig. 1b). All analyzed choriocarcinoma cell lines had a strong hypermethylation (50–100%) pattern of both promoter sections (Fig. 1b).

To investigate the influence of promoter methylation on gene expression we analyzed *RARRES1* expression by qRT-PCR (Fig. 1c). *RARRES1* mRNA expression was highest in primary isolated trophoblasts (12-fold compared to placental tissue), followed by the expression in Swan71 cells (5-fold) and total placental tissue (P). Corresponding to their methylation pattern, all choriocarcinoma cell lines showed a significantly lower *RARRES1* expression compared to placental tissue (*p* < 0.005), primary trophoblasts and the Swan71 cell line (Fig. 1c). To verify the correlation between increased CpG region 1 methylation and decreased *RARRES1* gene expression, we treated the cell lines with 0.5 μM (BeWo, JAR, *n* = 9) or 1.0 μM (Jeg-3, Swan71, *n* = 9) AZA for a time period of 72 h (Fig. 1d). Pyrosequencing of DNA from AZA treated cells revealed a significant decrease of CpG region 1 methylation in Jeg-3 and JAR cell lines (Additional file 4: Figure S2). *RARRES1* expression was significantly increased by AZA treatment in all choriocarcinoma cell lines (100- to 1200-fold, *p* < 0.005, Fig. 1d). In the Swan71 cell line *RARRES1* gene expression was about 2.5-fold higher following AZA-treatment when compared to untreated control (Fig. 1d).

### Induction of *RARRES1* expression by retinoic acid stimulation

**Fig. 2:**
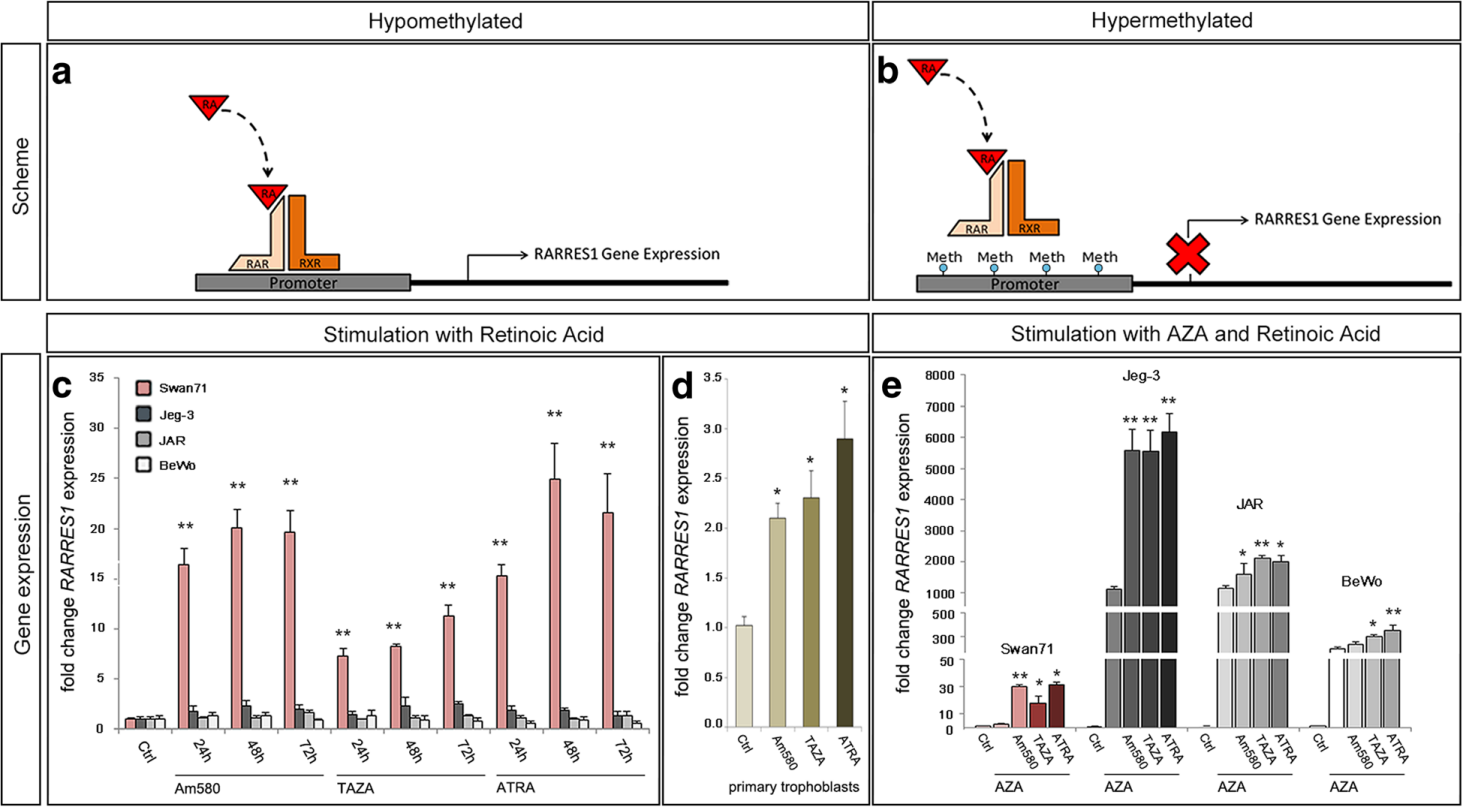
Retinoic acid induced *RARRES1* expression. **a** Scheme of Retinoic acid (RA) binding to Retinoic acid receptor (RAR and RXR) dimers. **b** Transcriptional activation of *RARRES1* expression is blocked by promoter methylation (Meth). **c** Swan71, Jeg-3, JAR and BeWo cells were treated with Am580, TAZA and ATRA for 24 to 72 h. **d** Primary trophoblasts (from *n* = 4 individuals) were treated with Am580, TAZA and ATRA for 48 h. Significance in (**c**) and (**d**) was calculated relative to untreated control cells (Ctrl.). **e** Cells were stimulated with Am580, TAZA and ATRA after AZA-treatment. Significance was determined in comparison to solely AZA-treated cells. Expressions of (**c**-**e**) are relative to Ctrl. Significance is marked by asterisks (* *p* < 0.05, ** *p* < 0.005). Data represent mean ± SEM

*RARRES1* gene expression is known to be upregulated by *RAR*-specific RAs in different tissues (Fig. 2a) [6]. Thus we investigated the effect of different RA derivates (Am580, TAZA and ATRA) stimulation on *RARRES1* gene expression in the choriocarcinoma cell lines and the first trimester cell line Swan71. ATRA is a RA which binds to all *RARs* (α, β and γ), whereas Am580 specifically binds to *RARα* [20]. TAZA is a *RARβ* and *γ* specific agonist [6]. Interestingly, a significant induction of *RARRES1* expression could only be achieved in the Swan71 cell line by Am580 (20-fold after 48 h), TAZA (17-fold after 72 h) and ATRA (35-fold after 48 h), with ATRA as the strongest agonist (Fig. 2c). On the other hand stimulation of the choriocarcinoma cell lines did not result in a significant increase of *RARRES1* expression (Fig. 2c). Furthermore, we stimulated primary trophoblasts with Am580, TAZA and ATRA for 48 h. We observed a significant induction of *RARRES1* expression of approximately 3.0-fold compared to untreated cells (Fig. 2d). We hypothesized that the lack of RA-responsiveness in choriocarcinoma cell lines might be due to the high methylation pattern of the *RARRES1* promoter (Fig. 1b and Fig. 2b). For verification, we additionally treated the choriocarcinoma cell lines with AZA for 72 h prior and during stimulation with *RAR*-specific RAs. Demethylation by AZA treatment allowed significant induction of *RARRES1* gene expression by RA stimulation compared to solitary AZA exposure (Fig. 2e). In Jeg-3 cells *RARRES1* expression was increased by 5-fold with Am580, TAZA and ATRA treatment. *RARRES1* expression of Jeg-3 cells after AZA and ATRA treatment was as high as *RARRES1* expression in untreated Swan71 cells. Increased *RARRES1* expression could also be observed for JAR and BeWo (1.4 to 1.8-fold and 1.2 to 1.8-fold, respectively; Fig. 2e). Treatment with AZA did not alter the RA response of Swan71 cells.

### Loss of *RARRES1* expression in choriocarcinoma tissue

**Fig. 3:**
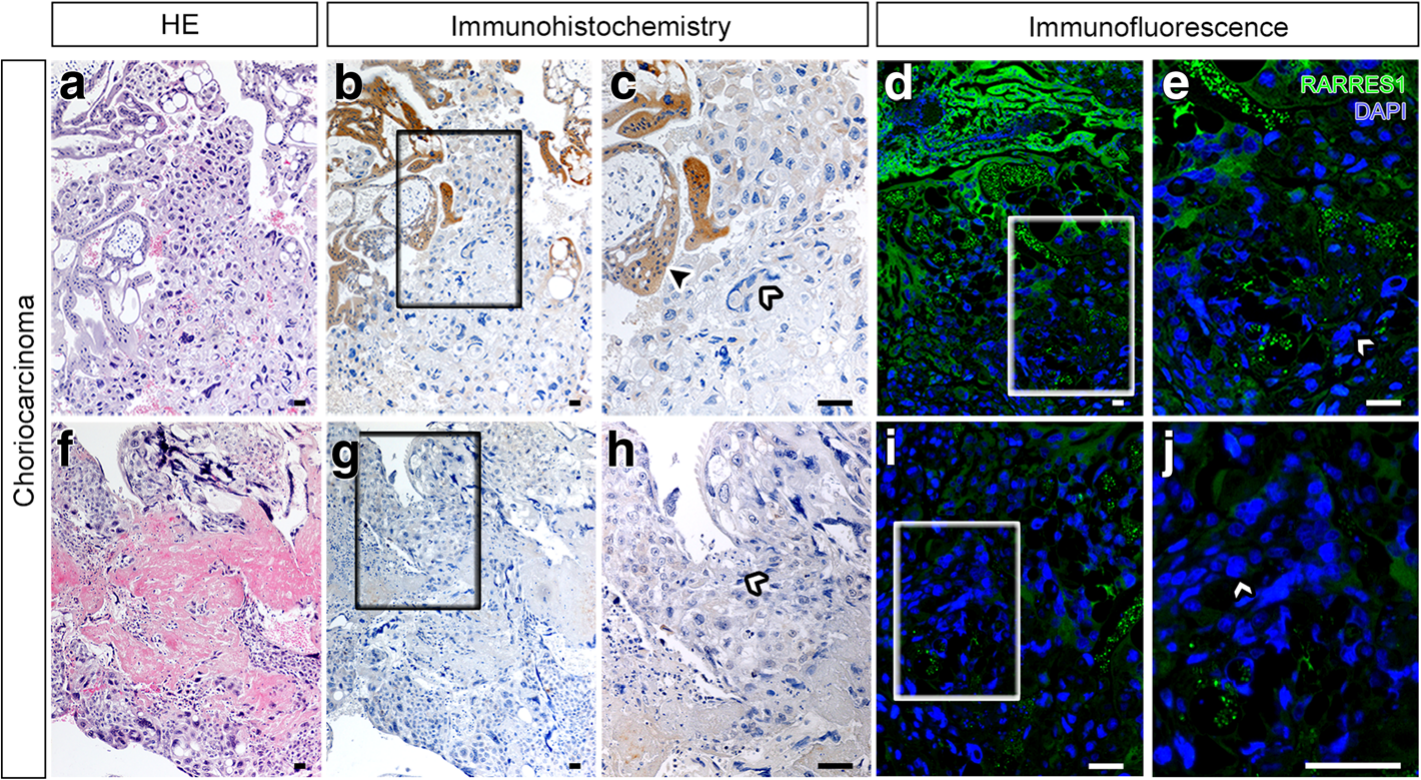
RARRES1 expression in choriocarcinoma sections. Sections of choriocarcinomas ((**a**-**e**) intramolare choriocarcinoma and (**f**-**j**) pure choriocarcinoma; *n* = 2 representative patients) were stained with anti-RARRES1 antibody by immunohistochemistry (**a**-**c** and **f**-**h**) or immunofluorescence (**d**, **e**, **i** and **j**). *RARRES1* positive staining is indicated in *brown* (**a**-**c** and **f**-**h**) or *green* (**d**, **e**, **i** and **j**), nuclei are *blue*. *RARRES1* positive cells are marked by *black arrow* heads; *white arrow* heads show *RARRES1* negative cells; (**c**, **e**, **h** and **j**) are magnifications of the sections in (**b**, **d**, **g** and **i**) marked by squares, respectively. The bar equals 100 μm

As shown earlier (Fig. 1d and Fig. 2c), hypermethylation of the *RARRES1* promoter was observed in choriocarcinoma cell lines and correlated with reduced gene expression and loss of transcriptional activation of *RARRES1*. In the light of these results we analyzed the RARRES1 expression in choriocarcinoma tissue (*n* = 10). Immunohistochemical and immunofluorescence staining of choriocarcinoma sections (intramolare a-e and pure f-j) revealed a loss of RARRES1 protein in malignant cancer cells (Fig. 3, white arrow heads). In contrast, cells of the molar trophoblast showed a strong RARRES1 positivity (Fig. 3c; black arrow head). Fig. 3 shows two choriocarcinoma cases (a-e and f-j) that were representative for the other eight analyzed tissues (data not shown).

### Correlation of *RARRES1* expression with cell density

**Fig. 4:**
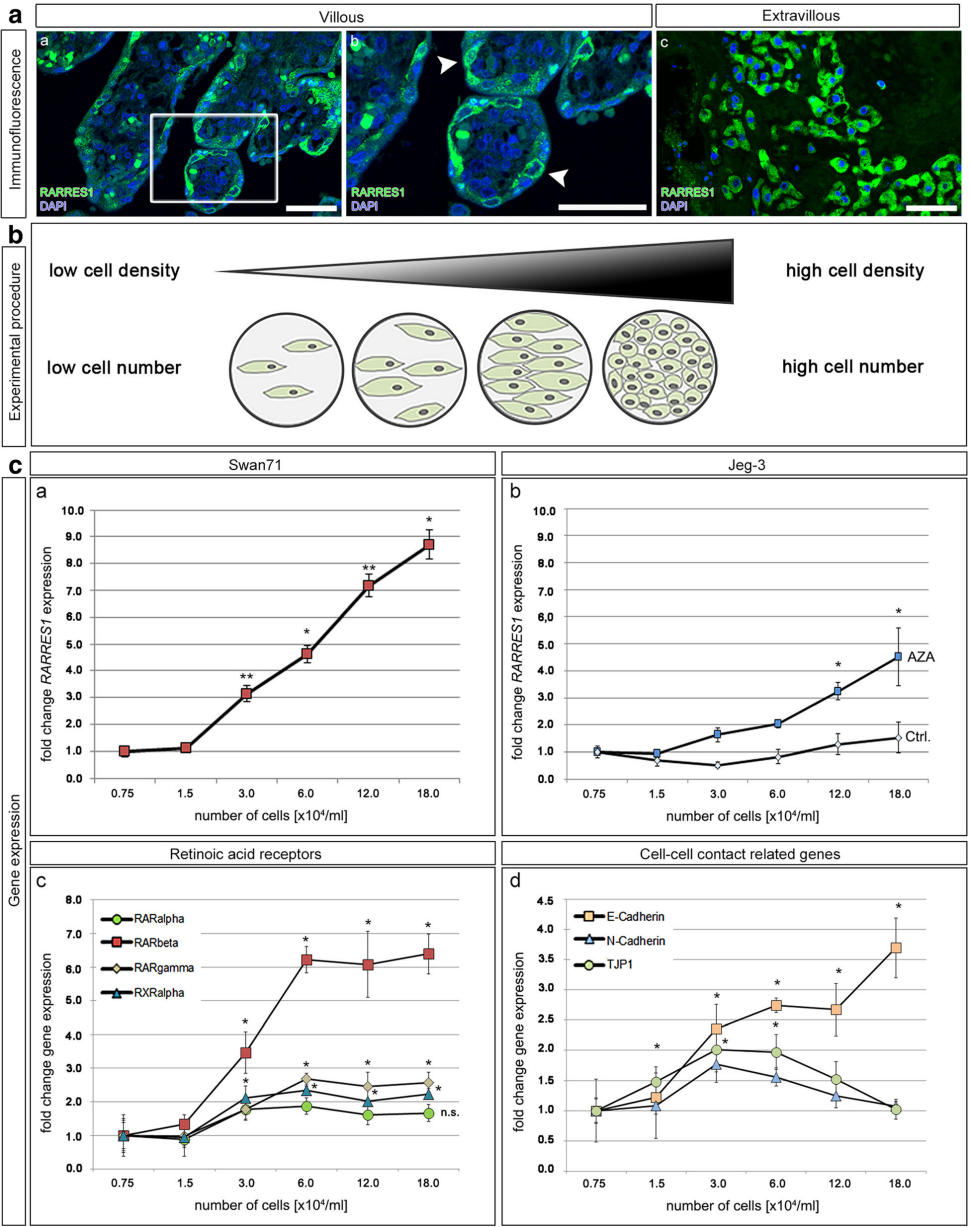
Correlation of cell density and *RARRES1* expression. **a** RARRES1 expression was analyzed in human third trimester placental sections by immunofluorescence staining. RARRES1 positivity is indicated by *green*. Nuclei are *blue*. **a** (*a*-*b)* represent the villous, *c* the extravillous part. *b* is a magnification of the square in *a*. *White arrow* heads indicate RARRES1 positive VTs, which are in cell-cell contact with the SCT. The bars equal 100 μm. **b** Scheme of the experimental procedure. Swan71 cells were seeded at different cell densities and RNA was isolated after 48 h. By qRT-PCR we compared the expression pattern of different genes in dependence of low to high cell densities normalized to 18SrRNA. **c** qRT-PCR analysis of *RARRES1* (*a* and *b*), *RARα, β, γ* and *RXRα* (**c**) as well as *E-Cadherin*, *N-Cadherin* and *TJP1* (*d*) expression in Swan71 (*a*, *c* and *d*) and Jeg-3 (*b*) cells. Gene expression was normalized to 18 s rRNA expression and set relative to the expression level observed at 0.75x10^4^ cells per ml. Significance is marked by asterisks (* *p* < 0.05, ** *p* < 0.005) and calculated relative to the expression at a cell density of 0.75*10^4^/ml. Data represent mean ± SEM

As *RARRES1* is downregulated in choriocarcinoma and significantly higher expressed in primary trophoblasts, we hypothesized that *RARRES1* might be of importance for normal trophoblastic development. By immunofluorescence staining of placental sections, we observed a prominent RARRES1 expression especially in VTs attached to the SCT (Fig. 4**a** and **b**; white arrow heads). Additionally, extravillous trophoblasts (EVTs) were RARRES1 positive (Fig. 4**a**, **c**). In order to analyze whether cell-cell connectivity influences *RARRES1* expression, we plated Swan71 and Jeg-3 cells in different cell densities, ranging from 0.75 to 18.0x10^4^ cells /ml (Additional file 2: Figure S1), and analyzed *RARRES1* gene expression normalized to 18SrRNA (Fig. 4**b** and **c**). We observed a significant increase of *RARRES1* expression with increasing Swan71 cell number per well (*n* = 6, Fig. 4**c**, **a**). High cell-cell contact rates, as observed in cell numbers above 6x10^4^/ml, were related with a significantly (*p* < 0.005) higher *RARRES1* expression (8.5-fold higher at 18.0x10^4^/ml compared to 0.75x10^4^/ml). In Jeg-3 cells the *RARRES1* expression did not change with increasing cell density (Fig. 4**c**, **b**). We hypothesized that this effect might be related to *RARRES1* promoter hypermethylation. Thus, we additionally treated Jeg-3 cells with AZA and analyzed *RARRES1* gene expression in dependence of cell density. After AZA treatment we detected a significant increase of *RARRES1* expression (*p* < 0.05, 4.5-fold at 18.0x10^4^ cells /ml) with rising cell numbers (Fig. 4**c**, b). In order to analyze the potential role of RARRES1 in cell connectivity we further applied real-time Electric Cell-substrate Impedance Sensing (ECIS) to RARRES1 overexpressing and Mock transfected Jeg-3 cells (Additional file 5). We observed a trend of increased impedance of confluent Jeg-3 cells overexpressing RARRES1 compared to Mock control (Additional file 6: Figure S3). Furthermore, RARRES1 or Mock transfected cells showed no difference in proliferation or migration (Additional file 5 and Additional file 6: Figure S3).

In order to investigate whether other genes involved in RA signaling were additionally upregulated with increasing cell density, we analyzed gene expression of the transcription factors *RARα*, *β*, *γ* and *RXRα* (Fig. 4**c**, c). We detected a significant increase of *RARβ*, *γ* and *RXRα* expression (Fig. 4**c**, c, *p* < 0.05), with *RARβ* exhibiting the most prominent increase (6.1-fold). Additionally, we analyzed the expression pattern of the epithelial and mesenchymal marker genes *E*- and *N-Cadherin* and the tight junction protein 1 (*TJP1*) (Fig. 4**c**, d). *E-Cadherin* increased significantly with rising cell density, while *N-Cadherin* expression increased up to 2.0-fold at a density of 3.0x10^4^/ml but declined at densities over 6.0x10^4^ cells/ml (Fig. 4**c**, d). Similarly, we observed a small increase of *TJP1* expression at 3.0x10^4^ cells/ml, which decreased with rising cell numbers (Fig. 4**c**, d). We further analyzed gene expression of *RARβ*, *E-Cadherin*, *N-Cadherin* and *TJP1* in Jeg-3 and JAR cells with siRNA-induced knock-down of *RARRES1* and *RARRES1* overexpression using a pDest-26 plasmid. We did not detect any differences regarding the gene expression compared to mock controls (data not shown).

### High RARRES1 expression in apoptotic and proliferative cells

**Fig. 5:**
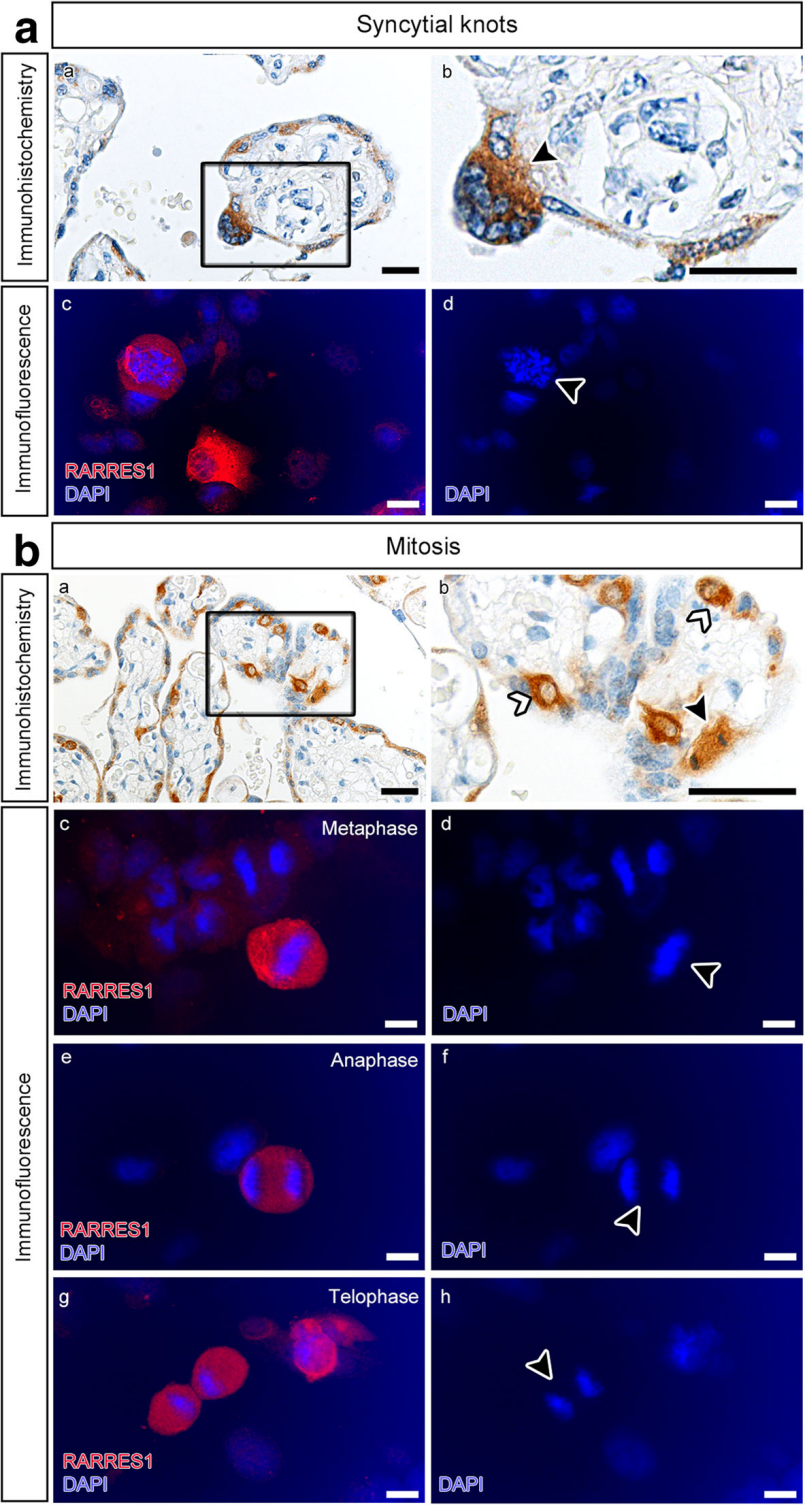
RARRES1 expression in placental tissue and Swan71 cells. **a** RARRES1 localization in syncytial knots and apoptotic cells was analyzed by immunohistochemistry of placental sections (*a* and *b*) and immunofluorescence of fixed Swan71 cells (*c* and *d*). RARRES1 positivity is indicated by *brown* (*a* and *b*) or *red* (*c*) staining. Nuclei are *blue* (*a*-*d*). **b** RARRES1 expression in dividing cells was analyzed by immunohistochemistry (*a* and *b*) of placental sections and immunofluorescence staining of Swan71 cells (*c*-*h*). *Black arrow* heads mark a VT during mitosis (*b*). Swan71 cells showed strong RARRES1 positivity during meta- (*c* and *d*), ana- (*e* and *f*) and telophase (*g* and *h*) of mitosis. The bars equal 100 μm

In order to characterize specific RARRES1 expression in human placental tissue and trophoblastic cell lines, we performed immunohistochemical and immunofluorescence staining (Fig. 5**a** and **b**). We especially detected high RARRES1 expression within syncytial knots (Fig. 5**a**, a and b). We further observed high RARRES1 expression in silenced cells with fragmented nuclei by immunofluorescence staining of Swan71 (Fig. 5**a**, c). Additionally, dividing cells showed a strong RARRES1 expression in placental tissue (Fig. 5**b**, a and b) as well as in Swan71 cells (Fig. 5**b**, c-h). RARRES1 expression was especially high during the meta-, ana- and telophase of proliferating Swan71 cells (Fig. 5**b**, c-h).

## Discussion

Taking the role of *RARRES1* as a tumor suppressor with a high methylation pattern in a variety of cancer types into consideration, we predicted that the *RARRES1* expression matched promoter methylation in placental tissues and cell lines [9, 21–23]. DNA from 3rd trimester placental tissues showed a low methylation pattern of CpG region 1 compared to a significantly higher overall methylation of CpG region 2. Consequently, we assumed CpG region 2 of the *RARRES1* promotor might be less relevant for RA-induced transcriptional activation compared to CpG region 1. In all observed choriocarcinoma cell lines both analyzed CpG regions were hypermethylated, which was accompanied by a significant decrease of *RARRES1* mRNA expression.

One limitation of the provided results is that AZA treatment of primary trophoblasts could not be performed due to the lack of proliferative capacity of third trimester trophoblasts. Trophoblasts of the third trimester undergo a terminal differentiation leading to an irreversible exit from the cell cycle and thus, AZA treatment of primary trophoblasts would not originate in the incorporation of 5′-AZA-2-dC into the DNA [24]. However, treatment of choriocarcinoma cell lines with AZA abolished the effect of high methylation resulting in an increased *RARRES1* gene expression. Nevertheless, we cannot rule out that increase of *RARRES1* expression was due to secondary side-effects, e.g. AZA-induced increase of transcription factors. It is well known that genes involved in RA-signaling are commonly methylated in cancer and that their expression can be induced by AZA treatment [19, 25]. For example, methylation of *RARRES1* correlates with *RARβ* promoter methylation in prostate cancer and treatment of colon and breast carcinoma cells with AZA induced a demethylation of the *RARβ* promoter region and a restoration of *RARβ* expression [19, 26, 27].

Regardless of secondary side-effects, we hypothesize that *RARRES1* expression and functional activity might be lost in choriocarcinomas, supporting the concept that placental RARRES1 might act in a suppressive manner and in cases of choriocarcinoma as a tumor suppressor. This is in line with our observation that RARRES1 protein expression in the intramolar choriocarcinoma tissue is limited to cells of the molar trophoblast. In comparison to the choriocarcinoma cell lines BeWo, JAR and Jeg-3, the first-trimester trophoblast-like cell line Swan71 showed a hypomethylation pattern at CpG region 1, while CpG region 2 was hypermethylated as well. Unlike Jeg-3, JAR and BeWo, this cell line does not originate from choriocarcinoma tissue. Swan71 is a telomerase immortalized cell line that is used as a model for first trimester trophoblasts [15]. In Swan71 cells the methylation of the entire *RARRES1* promoter was similar to the methylation pattern observed in placental tissues and isolated trophoblasts of the third trimester.

Peng et al. [9] previously analyzed *RARRES1* promoter methylation (up to 600 bp upstream of the ATG codon) in primary breast tumors along with matched adjacent benign tissues. In these patients, the methylation pattern of CpG region 1 (~200 bp upstream of the ATG codon) of the benign tissues was comparable to the pattern we observed in healthy placental tissue. Their data showed a low-to-high methylation pattern with increasing distance to the start codon of *RARRES1*. Even though Peng et al. solely investigated methylation up to 600 bp upstream from the ATG, this increase of methylation resembles the higher methylation pattern at CpG region 2 (>1000 bp upstream) observed by us. As CpG region 1 was hypomethylated in our placental tissues and subsequently open for transcriptional regulation, we hypothesized that the promoter area around CpG region 1 could be responsible for maintenance of *RARRES1* gene expression and that hypomethylation of this region might be essential for transcriptional regulation of *RARRES1* in trophoblasts*.*

With regard to functional aspects of RARRES1 expression, earlier publications showed that *RARRES1* is associated with a regulatory role in cancer invasiveness and tumorigenicity [5, 10]. For example, it is capable of suppressing the invasion and colony-forming ability of prostate cancer cells [10]. Here, we detected a high RARRES1 expression in VTs and EVTs and a loss of RARRES1 in malignant choriocarcinoma cells. Thus, we hypothesized that RARRES1 might influence trophoblast function during gestational development, possibly negatively regulating trophoblast invasion and/or fusion ability. Furthermore, our preliminary results in RARRES1 transfected Jeg-3 cells might indicate a potential role in cell connectivity.

Overall, RARRES1 is known to influence many cellular processes. It is able to interact with the transmembrane protein 192 (TMEM192) and thus induces the expression of autophagy related proteins [28]. Additionally, it was shown that RARRES1 affects the expression of valosine-containing protein (VCP), which induces the degradation of free polypeptides and thus is essential for autophagy, too [23, 29]. During human placental development autophagy is associated with the formation of syncytial knots and a reduced trophoblast invasion [30, 31]. This is in line with RARRES1 expression in syncytial knots and apoptotic cells seen by us. Consequently, we hypothesized that loss of RARRES1 in choriocarcinoma cells might induce an increased invasiveness and hinder autophagy as seen during malignant transformation [32]. On the other hand, RARRES1 is capable of inducing EB1 protein expression, which is essentially involved in the regulation of spindle dynamics and the spindle assembly checkpoint machinery [23, 33]. Furthermore, loss of both RARRES1 and EB1 protein expression is thought to be associated with the presence of cancer [33]. In Swan71 cells RARRES1 is strongly expressed during cell division (ana-, meta- and telophase). Thus, it might be of importance for cell cycle regulation in human trophoblasts and loss of protein expression might be associated with cell cycle deregulation in term of malignant transformation.

Another hallmark of cancer progression and especially metastasis is the epithelial to mesenchymal transition (EMT). In breast cancer cells RARRES1 was reported to interfere with beta-Catenin and AGBL2 function [34, 35]. Both proteins are involved in EMT of cancer cells or EVTs [35–38]. Expression of RARRES1 during the differentiation of EVTs to giant cells might thus be important to limit myometrial trophoblast invasiveness. On the other hand, loss of RARRES1 expression in choriocarcinoma cells might be essential for cancer cell invasion into the surrounding tissue. Additionally, we observed an induction of *RARRES1* and *RARβ* expression at high cell densities of Swan71 cells. *RARβ* is important for the transcriptional regulation of *RARRES1* expression and thus might be an essential inductor of high cell-density related *RARRES1* expression. In the light of high *RARRES1* expression in the SCT-neighboring VTs, we predict that *RARRES1* might also be involved in the regulation of cell-cell or cell-matrix contacts. As a tumor suppressor it might induce contact inhibition either through transmembrane connections or cell cycle regulation, and thus influence trophoblast behavior. Through its interaction with AGBL2, RARRES1 can inhibit the detyrosination of α-Tubulin [39]. α-Tubulin detyrosination is an important hallmark of EMT [40]. An increase of detyrosinated α-Tubulin induces the formation of microtentacles, which promote the penetration of endothelial cell layers and thus are directly linked to cancer invasiveness and metastasis [41]. Additionally, beta-Catenin is essential for cell adhesion and increases during cancer cell EMT [42]. Loss of RARRES1 expression stimulates the nuclear localization of beta-Catenin, which leads to the dissociation of E-Cadherin/beta-Catenin/alpha-Catenin complexes [34]. This process is important for the induction of cancer cell EMT and might explain the loss of RARRES1 expression in choriocarcinoma cases. In addition to increased *RARRES1* expression with high cell densities, we also observed rising *E-Cadherin* expression. This underscores the possible connection between RARRES1 and the E-Cadherin/beta-Catenin/alpha-Catenin complex. Thus, the ability of RARRES1 to reduce invasiveness and tumorigenicity might be due to its properties as a molecule regulating cell-adhesion [19]. RARRES1-triggered enhanced cell-cell contact of cancer cells might mediate contact inhibition of cell proliferation as well as decreased invasiveness and a reduced migratory capability [43]. Future studies should consider the in-depth investigation of RARRES1/E-Cadherin signaling and its potential regulatory role for cell-cell contact.

## Conclusion

Our data demonstrated that RARRES1 expression is lost in choriocarcinoma cases due to promotor hypermethylation. RARRES1 protein expression analysis by immunohistochemistry allowed the discrimination of intramolar and pure choriocarcinoma cases. We further hypothesized that RARRES1 might be a negative regulator of EMT through induction of contact inhibition.

## Additional files

Additional file 1: Table S1. Clinical data. (DOCX 18 kb)

Additional file 2: Figure S1. Light microscope image of Swan71 and Jeg-3 cells in different cell densities. Swan71 and Jeg-3 cells were seeded at different cell densities and light microscope images were taken after 48 h. (TIFF 1281 kb)

Additional file 3: Table S2. Primers. (DOCX 19 kb)

Additional file 4: Figure S2. DNA methylation of choriocarcinoma cell lines following AZA treatment. Swan71 (A), Jeg-3 (B), JAR (C) and BeWo (D) cells were treated with DMSO or AZA for 72 h and DNA methylation of CpG region 1 was measured by pyrosequencing (*n* = 3, respectively). Each circle represents one biological replicate. The bars represent the median with 95% CI. Significance is marked by asterisks (** *p* < 0.005). (TIFF 1049 kb)

Additional file 5: Supplementary Material and Method. (DOC 23 kb)

Additional file 6: Figure S3. Functional Assays using Jeg-3 cells overexpressing RARRES1. Jeg-3 cells were transfected with a RARRES1 pDest26 plasmid or a Mock control and Electric Cell-substrate Impedance sensing (ECIS, *n* = 6) was performed 48 h after transfection (A). The normalized mean impedance over a time period of 24 h of RARRES1 and Mock transfected cells and a medium control is presented (A). Efficient transfection was controlled by Western Blot analysis (B) using a Goat anti-Human RARRES1 polyclonal antibody (R&D Systems, Wiesbaden; 40 kDa) and a Rabbit anti-Human GAPDH polyclonal antibody (Santa Cruz, Heidelberg; 37 kDa). Additionally, proliferation of RARRES1 pDest26 and Mock transfected cells (C) (n = 3, respectively) was measured over a time period of 72 h using the CyQuant NF Cell Proliferation Kit (Thermo Fisher, Darmstadt). Migration of RARRES1 pDest26 and Mock transfected cells (*n* = 4, respectively) was measured over a time period of 48 h by Scratch Assay (D). The normalized proportion of the cell-free gap was calculated using ImageJ (Wayne Rasband, Nat. Institute of Health, USA). (TIFF 298 kb)

## Abbreviations

ATRA: All-trans retinoic acid
EMT: Epithelial-mesenchymal transition
EVT: Extravillous trophoblast
FFPE: Formalin fixed paraffin embedded
IF: Immunofluorescence
IHC: Immunohistochemistry
P: Placental tissue
qRT-PCR: Quantitative Realtime-PCR
RAR: Retinoic acid receptor
RARRES1: Retinoic acid receptor responder 1
SCT: Syncytiotrophoblast
SEM: Standard error of the mean
TAZA: Tazarotene
Tr: Primary trophoblasts
VT: Villous trophoblast

## References

[1] Yagel S, Parhar RS, Jeffrey JJ, Lala PK (1988). Normal nonmetastatic human trophoblast cells share in vitro invasive properties of malignant cells. J Cell Physiol.

[2] Fahlbusch FB, Ruebner M, Huebner H, Volkert G, Zuern C, Thiel F, Koch M, Menendez-Castro C, Wachter DL, Hartner A, Rascher W (2013). The tumor suppressor gastrokine-1 is expressed in placenta and contributes to the regulation of trophoblast migration. Placenta.

[3] Ferretti C, Bruni L, Dangles-Marie V, Pecking AP, Bellet D (2007). Molecular circuits shared by placental and cancer cells, and their implications in the proliferative, invasive and migratory capacities of trophoblasts. Hum Reprod Update.

[4] Coyle K, Murphy J, Vidovic D, Vaghar-Kashani A, Dean C, Sultan M, Clements D, Wallace M, Thomas M, Hundert A. Breast cancer subtype dictates DNA methylation and ALDH1A3-mediated expression of tumor suppressor RARRES1. Oncotarget. 2016; 10.18632/oncotarget.9858 PMC5190082 27286452

[5] Jing C, El-Ghany MA, Beesley C, Foster CS, Rudland PS, Smith P, Ke Y (2002). Tazarotene-induced gene 1 (TIG1) expression in prostate carcinomas and its relationship to tumorigenicity. J Natl Cancer Inst.

[6] Nagpal S, Patel S, Asano AT, Johnson AT, Duvic M, Chandraratna RA (1996). Tazarotene-induced gene 1 (TIG1), a novel retinoic acid receptor-responsive gene in skin. J Invest Dermatol.

[7] Kwong J, Lo KW, Chow LSN, Chan FL, Huang DP, To KF (2005). Silencing of the retinoid response gene TIG1 by promoter hypermethylation in nasopharyngeal carcinoma. Int J Cancer.

[8] Kloth M, Goering W, Ribarska T, Arsov C, Sorensen KD, Schulz WA (2012). The SNP rs6441224 influences transcriptional activity and prognostically relevant hypermethylation of RARRES1 in prostate cancer. Int J Cancer.

[9] Peng Z, Shen R, Li Y-W, Teng K-Y, Shapiro CL, Lin H-JL (2012). Epigenetic repression of RARRES1 is mediated by methylation of a proximal promoter and a loss of CTCF binding. PLoS One.

[10] Oldridge EE, Walker HF, Stower MJ, Simms MS, Mann VM, Collins AT, Pellacani D, Maitland NJ (2013). Retinoic acid represses invasion and stem cell phenotype by induction of the metastasis suppressors RARRES1 and LXN. Oncogene.

[11] Hui P, Martel M, Parkash V (2005). Gestational trophoblastic diseases: recent advances in histopathologic diagnosis and related genetic aspects. Adv Anat Pathol.

[12] Wong NC, Novakovic B, Weinrich B, Dewi C, Andronikos R, Sibson M, Macrae F, Morley R, Pertile MD, Craig JM, Saffery R (2008). Methylation of the adenomatous polyposis coli (APC) gene in human placenta and hypermethylation in choriocarcinoma cells. Cancer Lett.

[13] Novakovic B, Rakyan V, Ng HK, Manuelpillai U, Dewi C, Wong NC, Morley R, Down T, Beck S, Craig JM, Saffery R (2008). Specific tumour-associated methylation in normal human term placenta and first-trimester cytotrophoblasts. Mol Hum Reprod.

[14] Ruebner M, Strissel PL, Langbein M, Fahlbusch F, Wachter DL, Faschingbauer F, Beckmann MW, Strick R (2010). Impaired cell fusion and differentiation in placentae from patients with intrauterine growth restriction correlate with reduced levels of HERV envelope genes. J Mol Med (Berl).

[15] Straszewski-Chavez SL, Abrahams VM, Alvero AB, Aldo PB, Ma Y, Guller S, Romero R, Mor G (2009). The isolation and characterization of a novel telomerase immortalized first trimester trophoblast cell line, swan 71. Placenta.

[16] Ruebner M, Strissel PL, Ekici AB, Stiegler E, Dammer U, Goecke TW, Faschingbauer F, Fahlbusch FB, Beckmann MW, Strick R (2013). Reduced syncytin-1 expression levels in placental syndromes correlates with epigenetic hypermethylation of the ERVW-1 promoter region. PLoS One.

[17] Li L-C, Dahiya R (2002). MethPrimer: designing primers for methylation PCRs. Bioinformatics.

[18] Tost J, El Abdalaoui H, Gut IG (2006). Serial pyrosequencing for quantitative DNA methylation analysis. BioTechniques.

[19] Zhang J, Liu L, Pfeifer GP (2003). Methylation of the retinoid response gene TIG1 in prostate cancer correlates with methylation of the retinoic acid receptor beta gene. Oncogene.

[20] Bosch A, Bertran SP, Lu Y, Garcia A, Jones AM, Dawson MI, Farias EF (2012). Reversal by RARα agonist Am580 of c-Myc-induced imbalance in RARα/RARγ expression during MMTV-Myc tumorigenesis. Breast Cancer Res.

[21] Wu CC, Shyu RY, Chou JM, Jao SW, Chao PC, Kang JC, Wu ST, Huang SL, Jiang SY (2006). RARRES1 expression is significantly related to tumour differentiation and staging in colorectal adenocarcinoma. Eur J Cancer.

[22] Kwok WK, Pang JC, Lo KW, Ng HK (2009). Role of the RARRES1 gene in nasopharyngeal carcinoma. Cancer Genet Cytogenet.

[23] Sahab ZJ, Hall MD, Zhang L, Cheema AK, Byers SW (2010). Tumor suppressor RARRES1 regulates DLG2, PP2A, VCP, EB1, and Ankrd26. J Cancer.

[24] Soundararajan R, Rao AJ (2004). Trophoblast 'pseudo-tumorigenesis': significance and contributory factors. Reprod Biol Endocrinol.

[25] Shutoh M, Oue N, Aung PP, Noguchi T, Kuraoka K, Nakayama H, Kawahara K, Yasui W (2005). DNA methylation of genes linked with retinoid signaling in gastric carcinoma. Cancer.

[26] Yang Q, Shan L, Yoshimura G, Nakamura M, Nakamura Y, Suzuma T, Umemura T, Mori I, Sakurai T, Kakudo K (2002). 5-aza-2′-deoxycytidine induces retinoic acid receptor beta 2 demethylation, cell cycle arrest and growth inhibition in breast carcinoma cells. Anticancer Res.

[27] Cote S, Sinnett D, Momparler RL (1998). Demethylation by 5-aza-2′-deoxycytidine of specific 5-methylcytosine sites in the promoter region of the retinoic acid receptor beta gene in human colon carcinoma cells. Anti-Cancer Drugs.

[28] Shyu R, Wang C, Wu C, Chen M, Lee M, Wang L, Jiang S, Tsai F. Tazarotene-induced gene 1 enhanced cervical cell Autophagy through Transmembrane protein 192. Mol cells. 2016; 10.14348/molcells.2016.0161 PMC5223105 27989102

[29] Ju J-S, Fuentealba RA, Miller SE, Jackson E, Piwnica-Worms D, Baloh RH, Weihl CC (2009). Valosin-containing protein (VCP) is required for autophagy and is disrupted in VCP disease. J Cell Biol.

[30] Gong J-S, Kim GJ (2014). The role of autophagy in the placenta as a regulator of cell death. Clin exp reprod med.

[31] Heazell A, Moll S, Jones C, Baker P, Crocker I (2007). Formation of syncytial knots is increased by hyperoxia, hypoxia and reactive oxygen species. Placenta.

[32] Galluzzi L, Pietrocola F, Bravo-San Pedro JM, Amaravadi RK, Baehrecke EH, Cecconi F, Codogno P, Debnath J, Gewirtz DA, Karantza V (2015). Autophagy in malignant transformation and cancer progression. EMBO J.

[33] Green RA, Wollman R, Kaplan KB (2005). APC and EB1 function together in mitosis to regulate spindle dynamics and chromosome alignment. Mol Biol Cell.

[34] Tsai F-M, Wu C-C, Shyu R-Y, Wang C-H, Jiang S-Y (2011). Tazarotene-induced gene 1 inhibits prostaglandin E2-stimulated HCT116 colon cancer cell growth. J Biomed Sci.

[35] Sahab ZJ, Hall MD, Sung YM, Dakshanamurthy S, Ji Y, Kumar D, Byers SW (2011). Tumor suppressor RARRES1 interacts with cytoplasmic carboxypeptidase AGBL2 to regulate the α-tubulin tyrosination cycle. Cancer Res.

[36] Li HW, Cheung AN, Tsao SW, Cheung AL (2003). O WS: expression of e-cadherin and beta-catenin in trophoblastic tissue in normal and pathological pregnancies. Int J Gynecol Pathol.

[37] Candelier J-J, Frappart L, Diatta AL, Yadaden T, Cissé M-L, Afoutou J-M, Picard J-Y, Mennesson B, Slim R, Si-Tayeb K (2013). Differential expression of E-cadherin, β-catenin, and Lewis x between invasive hydatidiform moles and post-molar choriocarcinomas. Virchows Arch.

[38] Rahnama F, Shafiei F, Gluckman PD, Mitchell MD, Lobie PE (2006). Epigenetic regulation of human trophoblastic cell migration and invasion. Endocrinology.

[39] Sahab ZJ, Hall MD, Me Sung Y, Dakshanamurthy S, Ji Y, Kumar D, Byers SW: Tumor suppressor RARRES1 interacts with cytoplasmic carboxypeptidase AGBL2 to regulate the alpha-tubulin tyrosination cycle. Cancer Res 2011, 71:1219-1228. 10.1158/0008-5472.CAN-10-2294 PMC3062087 21303978

[40] Whipple RA, Matrone MA, Cho EH, Balzer EM, Vitolo MI, Yoon JR, Ioffe OB, Tuttle KC, Yang J, Martin SS (2010). Epithelial-to-mesenchymal transition promotes tubulin detyrosination and microtentacles that enhance endothelial engagement. Cancer Res.

[41] Korb T, Schlüter K, Enns A, Spiegel H-U, Senninger N, Nicolson GL, Haier J (2004). Integrity of actin fibers and microtubules influences metastatic tumor cell adhesion. Exp Cell Res.

[42] Brembeck FH, Rosário M, Birchmeier W (2006). Balancing cell adhesion and Wnt signaling, the key role of β-catenin. Curr Opin Genet Dev.

[43] Baba M, Kishida T, Yao M, Ohno S, Baba M (2001). Tumor suppressor protein VHL is induced at high cell density and mediates contact inhibition of cell growth. Oncogene.

