# Supplementary material for: Hypermethylation and loss of retinoic acid receptor responder 1 expression in human choriocarcinoma
Source: J Exp Clin Cancer Res. 2017 Nov 23;36:165. doi: 10.1186/s13046-017-0634-x (PMC5701501; doi:10.1186/s13046-017-0634-x)
Supplement: Supplementary file 1 — Clinical data. (DOCX 18 kb) [file 13046_2017_634_MOESM1_ESM.docx]

**Additional file 1: Table S1 Clinical data**

| \|  \| P (n=74) \| \| --- \| --- \| \|  \| mean ± SEM \| \| Gravida \| 2.22 ± 0.15 \| \| Para \| 0.75 ± 0.11 \| \| Max. systolic blood pressure [mmHg] \| n.d. \| \| Max. diastolic blood pressure [mmHg] \| n.d. \| \| Gestational age [weeks] \| 36.92 ± 0.50 \| \| Birth weight [g] \| 2853.00 ± 113 \| \| Urinary protein [mg/24h] \| n.d. \| |  |  |  |  |  |  |
| --- | --- | --- | --- | --- | --- | --- | --- | --- | --- | --- | --- | --- | --- | --- | --- | --- | --- | --- | --- | --- | --- | --- | --- | --- |
| n.d. = not done |  |  |  |  |  |  |
