# Supplementary figures and images for: Hypermethylation and loss of retinoic acid receptor responder 1 expression in human choriocarcinoma
Source: J Exp Clin Cancer Res. 2017 Nov 23;36:165. doi: 10.1186/s13046-017-0634-x (PMC5701501; doi:10.1186/s13046-017-0634-x)

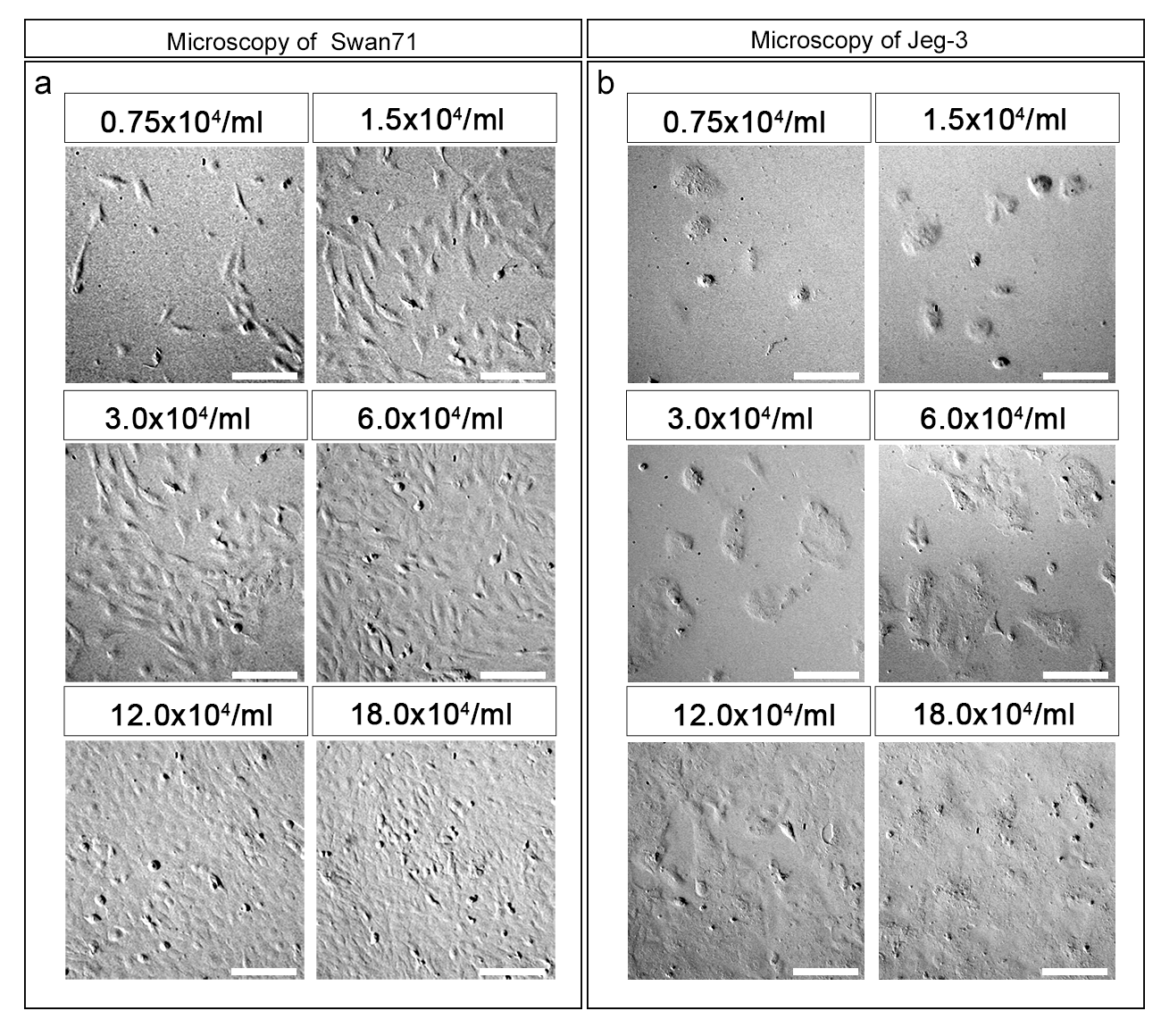

Supplement: Supplementary file 2 — Light microscope image of Swan71 and Jeg-3 cells in different cell densities. Swan71 and Jeg-3 cells were seeded at different cell densities and light microscope images were taken after 48 h. (TIFF 1281 kb) [file 13046_2017_634_MOESM2_ESM.tif]

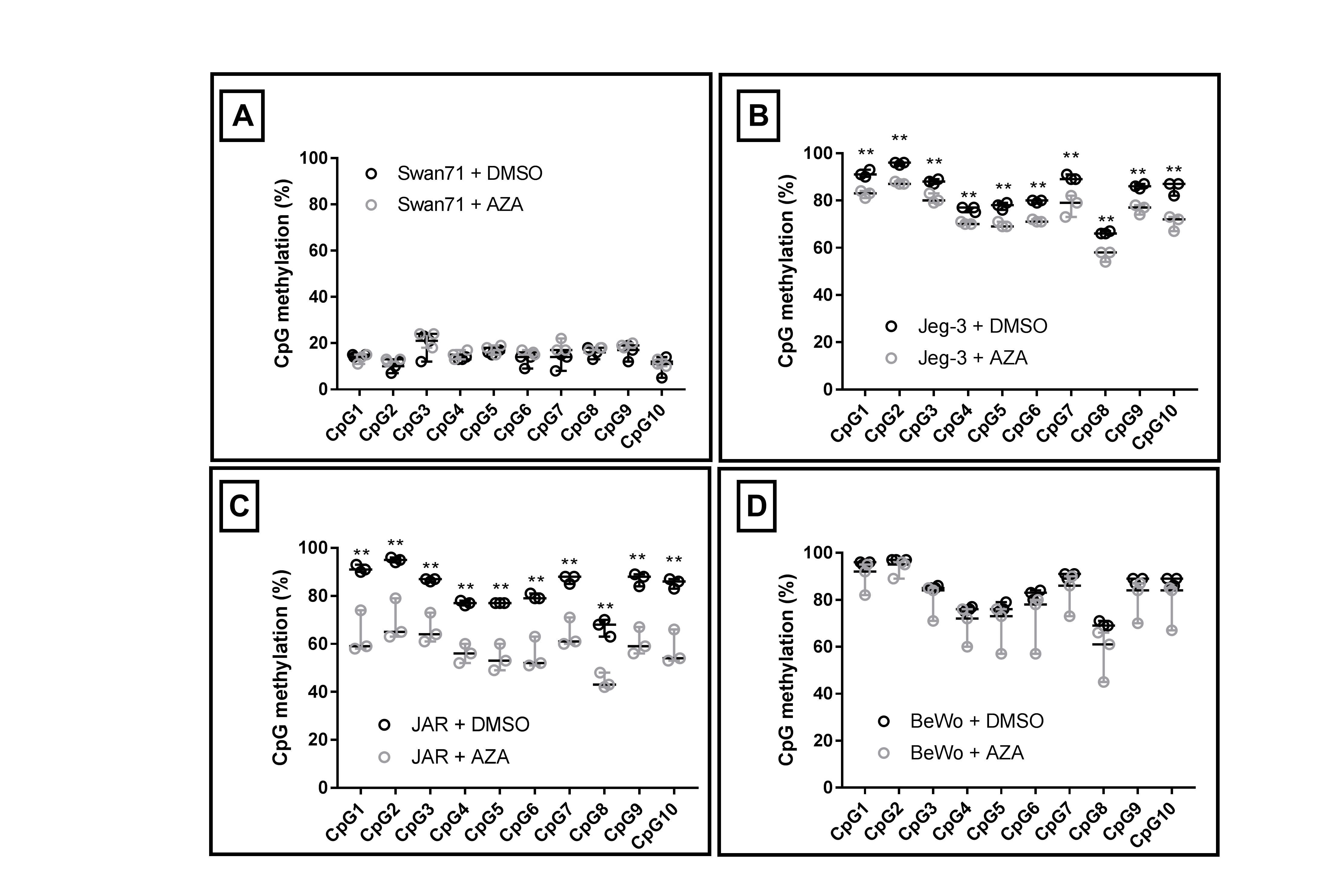

Supplement: Supplementary file 4 — DNA methylation of choriocarcinoma cell lines following AZA treatment. Swan71 (A), Jeg-3 (B), JAR (C) and BeWo (D) cells were treated with DMSO or AZA for 72 h and DNA methylation of CpG region 1 was measured by pyrosequencing (n = 3, respectively). Each circle represents one biological replicate. The bars represent the median with 95% CI. Significance is marked by asterisks (** p < 0.005). (TIFF 1049 kb) [file 13046_2017_634_MOESM4_ESM.tif]

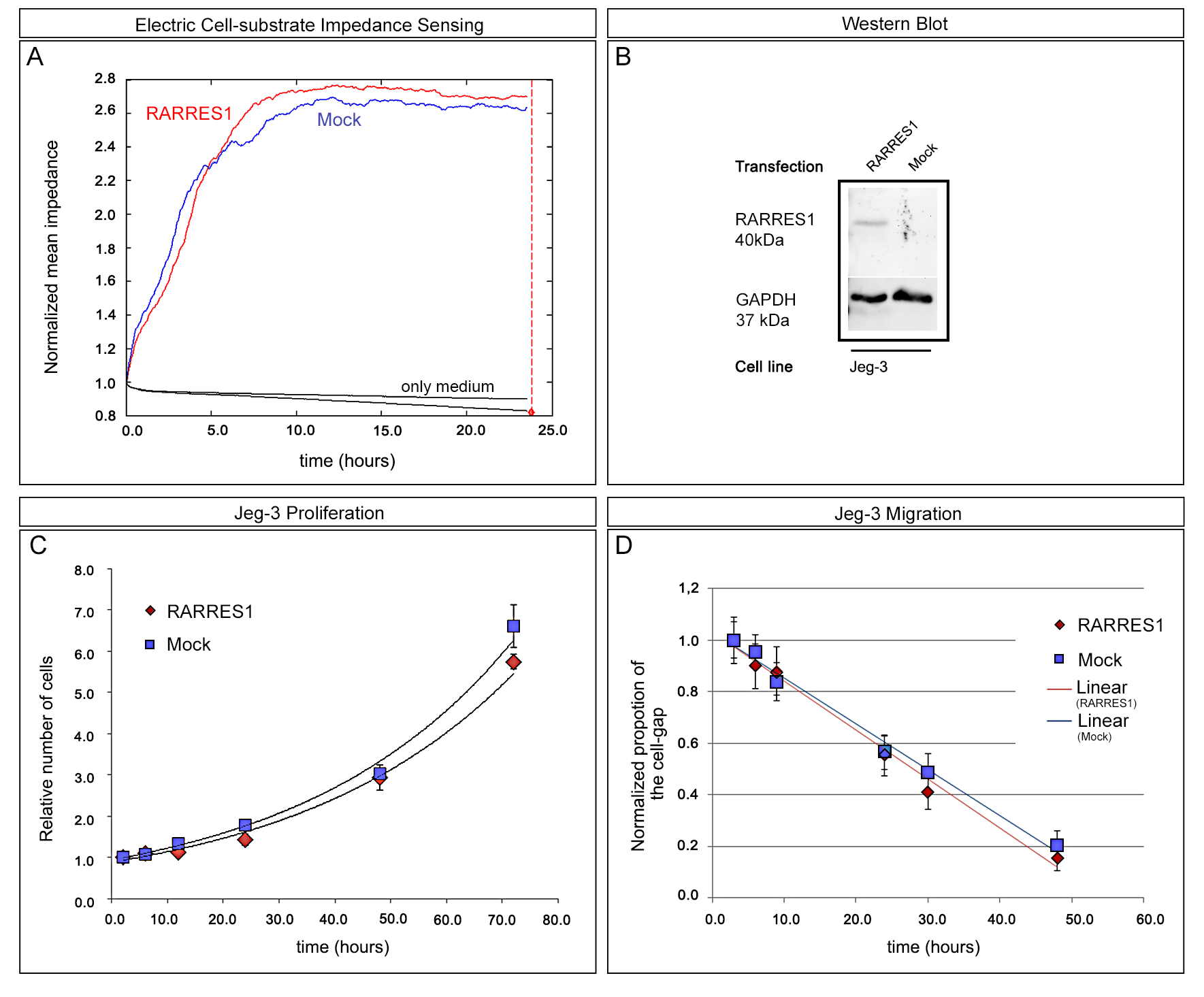

Supplement: Supplementary file 6 — Functional Assays using Jeg-3 cells overexpressing RARRES1. Jeg-3 cells were transfected with a RARRES1 pDest26 plasmid or a Mock control and Electric Cell-substrate Impedance sensing (ECIS, n = 6) was performed 48 h after transfection (A). The normalized mean impedance over a time period of 24 h of RARRES1 and Mock transfected cells and a medium control is presented (A). Efficient transfection was controlled by Western Blot analysis (B) using a Goat anti-Human RARRES1 polyclonal antibody (R&D Systems, Wiesbaden; 40 kDa) and a Rabbit anti-Human GAPDH polyclonal antibody (Santa Cruz, Heidelberg; 37 kDa). Additionally, proliferation of RARRES1 pDest26 and Mock transfected cells (C) (n = 3, respectively) was measured over a time period of 72 h using the CyQuant NF Cell Proliferation Kit (Thermo Fisher, Darmstadt). Migration of RARRES1 pDest26 and Mock transfected cells (n = 4, respectively) was measured over a time period of 48 h by Scratch Assay (D). The normalized proportion of the cell-free gap was calculated using ImageJ (Wayne Rasband, Nat. Institute of Health, USA). (TIFF 298 kb) [file 13046_2017_634_MOESM6_ESM.tif]
