# Supplementary material for: Hypermethylation and loss of retinoic acid receptor responder 1 expression in human choriocarcinoma
Source: J Exp Clin Cancer Res. 2017 Nov 23;36:165. doi: 10.1186/s13046-017-0634-x (PMC5701501; doi:10.1186/s13046-017-0634-x)
Supplement: Supplementary file 3 — Primers. (DOCX 19 kb) [file 13046_2017_634_MOESM3_ESM.docx]

**Additional file 3: Table S2 Primers**

|  | **Forward** | **Reverse** |
| --- | --- | --- |
| **qRT-PCR** |  |  |
| hu_Rarres1 | TTAATCCAAAAGAGGGATGTAAAGTTC | CGTCCCTCACCTTCCTGAAG |
| RARalpha | GATGCTAATGAAGATTACTGACCTG | CATTTCCTGGATGAGAGGCGG |
| RARbeta | ACACAGAAACAGGCCTTCTCAGT | AGCAATGGTTCTTGTAGCTTATCTAC |
| RARgamma | GCTACCAAGTGCATCATCAAGATC | CAGATACGCAGCATCAGGATATCT |
| RXRalpha | CCATCTTTGACAGGGTGCTGA | AGTCAGGGTTAAAGAGGACGATG |
| E-Cadherin | GCCTCAGAAGACAGAAGAGAGACT | GATCTGAACCAGGTTTTTAGGAAA |
| N-Cadherin | TGATCCTTATCGGTCACAGTTAGA | TCAATGACAATCCTCCAGAGTTTA |
| TJP | AGCACAGCAATGGAGGAAACA | TCCCCACTCTGAAAATGAGGA |
| 18srRNA | GCAATTATTCCCCATGAACG | GGCCTCACTAAACCATCCAA |
| GAPDH | CTCTGCTGTAGGCTCATTTGC | ACCAAAGTTGTCATGGATGACCT |
| **PCR** |  |  |
| CpG region 1 | biotin-TTTTGAATAAAATGTAGGATGGAGAA | CAAATTCAAACAATTCTACCTCAAC |
| CpG region 2 | biotin-AAGTTTTTGAGTTGGGTTAGGGA | TCCCCATAAAACCACTCCTTTTC |
|  | **1^st^ section** | **2^nd^ section** |
| **Sequencing** |  |  |
| CpG region 1 | AACCCCTAAATTCTCTCTAC | AATACTTCTAACCCAAAC |
| CpG region 2 | CTAATCTCAAACTCCTAACC | CCAAAATACTAAAATTACAA |
