# Supplementary material for: Hypermethylation and loss of retinoic acid receptor responder 1 expression in human choriocarcinoma
Source: J Exp Clin Cancer Res. 2017 Nov 23;36:165. doi: 10.1186/s13046-017-0634-x (PMC5701501; doi:10.1186/s13046-017-0634-x)
Supplement: Supplementary file 4 [file 13046_2017_634_MOESM5_ESM.doc]

Additional file 5

Electric Cell-substrate Impedance Sensing

For the measurement of impedance as a sign of cell-cell connectivity we used Electric Cell-substrate Impedance Sensing (ECIS, ibidi, Martinsreid). Jeg-3 cells were transfected with the RARRES1 pDest-26 (ImaGenes, Berlin) expression vector or a Mock control using jetPEI (PolyPlus, Illkirch, France) as transfection reagent. Cells were seeded into 8W1E plates at a density of 1.2x105cells/400µl. After shutting down an electric fence in the center of the dish, cells were able to grow for 24 hours. For the measurement of impedance a small current was applied at the electrode of the plates. The impedance was calculated by division of the measured potential by the alternating current. The impedance was determined at different frequencies in a time-dependent manner (Jiang WG. Electric cell-substrate impedance sensing and cancer metastasis. Springer Science & Business Media 2012).

Proliferation Assay

Jeg-3 cells were transfected with the RARRES1 pDest-26 (ImaGenes, Berlin) expression vector or a Mock control using jetPEI (PolyPlus, Illkirch, France) as transfection reagent. 48 hours after transfection cells were seeded in a 96 well plate (BD Biosciences, Heidelberg) at a density of 1,000 cells per well. Proliferation was measured after 2, 6, 12, 24, 48 and 72 hours using the CyQuant NF Cell Proliferation Kit (Thermo Fisher, Darmstadt). Fluorescence was detected using the GENios FL instrument (Tecan, Crailsheim).

Migration Assay

Jeg-3 cells were seeded in a 12 well plate at a density of 1.5x105/ml and transfected with the RARRES1 pDest-26 (ImaGenes, Berlin) expression vector or a Mock control using jetPEI (PolyPlus, Illkirch, France) as a transfection reagent (n=4, respectively). 48 hours after transfection a Scratch Assay was performed. Photos of 4 different areas per well were taken at 3, 6, 9, 24, 30 and 48 hours after a Scratch was drawn using a 200µl pipette tip. The cell-free area of the gap was measured using ImageJ (Wayne Rasband, Nat. Institute of Health, USA). 

Western Blot

Western Blot analysis was performed to determine RARRES1 overexpression after transfection of Jeg-3 cells. Cells were lysed using RIPA buffer. Protein concentration was determined using EZQ fluorescence based protein quantification (Thermo Fisher, Darmstadt). 20µg was loaded on a 10% SDS-agarose gel. After electrophoresis and transfer onto nitrocellulose membrane, the membrane was blocked with 2% non-fat milk for 30min at room temperature and incubated with the primary antibody (Goat anti-Human RARRES1 polyclonal, 1.0 µg/ml, R&D systems) at 4°C over night. As a secondary antibody Donkey anti-Goat was used (1:1000, Santa Cruz). Detection of the bands was performed using chemiluminescence (Genegnome, Syngene).
